# A genome-wide perspective about the diversity and demographic history of seven Spanish goat breeds

**DOI:** 10.1186/s12711-016-0229-6

**Published:** 2016-07-25

**Authors:** Arianna Manunza, Antonia Noce, Juan Manuel Serradilla, Félix Goyache, Amparo Martínez, Juan Capote, Juan Vicente Delgado, Jordi Jordana, Eva Muñoz, Antonio Molina, Vincenzo Landi, Agueda Pons, Valentin Balteanu, Amadou Traoré, Montse Vidilla, Manuel Sánchez-Rodríguez, Armand Sànchez, Tainã Figueiredo Cardoso, Marcel Amills

**Affiliations:** 1 Department of Animal Genetics, Center for Research in Agricultural Genomics (CSIC-IRTA-UAB-UB), Campus Universitat Autònoma de Barcelona, 08193 Bellaterra, Spain; 2 Departamento de Genética, Universidad de Córdoba, 14071 Córdoba, Spain; 3 Área de Genética y Reproducción Animal, SERIDA-Deva, Camino de Rioseco 1225, Gijón, 33394 Spain; 4 Instituto Canario de Investigaciones Agrarias, 38108 La Laguna, Tenerife Spain; 5 Departament de Ciència Animal i dels Aliments, Universitat Autònoma de Barcelona, 08193 Bellaterra, Spain; 6 Unitat de Races Autòctones, Servei de Millora Agrària i Pesquera (SEMILLA), 07198 Son Ferriol, Spain; 7 Faculty of Animal Science and Biotechnologies and Institute of Life Sciences, University of Agricultural Sciences and Veterinary Medicine, 400372 Cluj-Napoca, Romania; 8 Institut de l’Environnement et Recherches Agricoles, 04 BP 8645, Ouagadougou 04, Burkina Faso; 9 Associació de Ramaders de Cabra Blanca de Rasquera, Rasquera, 43513 Spain

## Abstract

**Background:**

The main goal of the current work was to infer the demographic history of seven Spanish goat breeds (Malagueña, Murciano-Granadina, Florida, Palmera, Mallorquina, Bermeya and Blanca de Rasquera) based on genome-wide diversity data generated with the Illumina Goat SNP50 BeadChip (population size, N = 176). Five additional populations from Europe (Saanen and Carpathian) and Africa (Tunisian, Djallonké and Sahel) were also included in this analysis (N = 80) for comparative purposes.

**Results:**

Our results show that the genetic background of Spanish goats traces back mainly to European breeds although signs of North African admixture were detected in two Andalusian breeds (Malagueña and Murciano-Granadina). In general, observed and expected heterozygosities were quite similar across the seven Spanish goat breeds under analysis irrespective of their population size and conservation status. For the Mallorquina and Blanca de Rasquera breeds, which have suffered strong population declines during the past decades, we observed increased frequencies of large-sized (ROH), a finding that is consistent with recent inbreeding. In contrast, a substantial part of the genome of the Palmera goat breed comprised short ROH, which suggests a strong and ancient founder effect.

**Conclusions:**

Admixture with African goats, genetic drift and inbreeding have had different effects across the seven Spanish goat breeds analysed in the current work. This has generated distinct patterns of genome-wide diversity that provide new clues about the demographic history of these populations.

**Electronic supplementary material:**

The online version of this article (doi:10.1186/s12711-016-0229-6) contains supplementary material, which is available to authorized users.

## Background

Since their domestication in the Fertile Crescent, goats are bred by humans as a source of milk, meat and fiber. Breeding has led to substantial changes in the patterns of genomic diversity of caprine breeds [1]. Intensive reproduction and genetic management practices have contributed to the decrease in effective size of goat populations as well as to increased inbreeding because of the small number of bucks used as breeders. Moreover, the spread of a few highly productive caprine breeds around the world and the progressive abandonment of low income rural activities have played a key role in the demographic recession, and even the extinction, of many local goat breeds. However, nomad pastoralism, trade and transhumance have favoured genetic exchanges between goat populations that are raised in distant locations, thus increasing their diversity and reducing the effects of genetic drift [2].

According to the classification criteria established by the Spanish Ministry of Environment, Rural and Marine Affairs [3], in Spain there are 22 officially recognized caprine breeds: five of them are classified as promotion breeds due to their economic importance and growing censuses and 17 have a status of special protection because of their demographic decline that, in some cases, was very severe. A number of these Spanish populations have been analysed with mitochondrial [4] and microsatellite markers [5, 6], which revealed high levels of variability and a weak population structure. However, due to the lack of population genomic studies, it is not possible to make inferences about the impact of evolutionary forces and demography on whole-genome variation. In the work presented here, our aim was to provide a high-resolution picture of the genome-wide diversity and population structure of seven Spanish goat breeds (Murciano-Granadina, Malagueña, Florida, Bermeya, Blanca de Rasquera, Palmera and Mallorquina) by genotyping 176 individuals with single nucleotide polymorphisms (SNPs) from the Goat SNP50 BeadChip [7]. Recent studies performed on cattle data have identified patterns of admixture between bovine breeds from Southern Europe and Africa [8], thus our second objective was to investigate if Spanish goats have been introgressed with African goat breeds. With this purpose, we genotyped 80 additional individuals from European (Saanen and Carpathian) and African (Tunisian, Djallonké and Sahel) reference populations.

## Methods

### Ethics statement

Sampling was performed by trained veterinarians in the context of sanitation campaigns and parentage controls that were not directly related with our research project. For this reason, it was not necessary to obtain permission from the Universitat Autònoma de Barcelona Committee of Ethics in Animal Experimentation. In all instances, veterinarians followed standard procedures and relevant national guidelines to ensure appropriate animal care

### Goat sampling and genotyping with the Goat SNP50 BeadChip

Blood samples were collected by jugular venipuncture in Vacutainer EDTA-containing tubes. We sampled 176 individuals from seven Spanish goat breeds (see Additional file 1: Table S1) that were selected according to the following criteria. First, since our aim was to compare goat breeds with divergent demographies, we chose three promotion breeds and four breeds with a special protection status (see Additional file 1: Table S1, Additional file 2: Figure S1). Second, breeds were also selected on the basis of their geographic distribution, i.e. populations were sampled from northern (Bermeya and Blanca de Rasquera) and southern (Florida, Malagueña, Murciano-Granadina) Spain to test if differential introgression with African breeds had occurred in goats from these two locations. Populations from insular territories (Palmera, Mallorquina) were also of interest because they provided the opportunity to test the genetic consequences of founder effects and geographic isolation on genome-wide diversity. Unrelated individuals were sampled on 2 to 13 farms to ensure a good representation of the diversity of each breed (see Additional file 1: Table S1). The numbers of males and females differed but we assumed that this would have little effect on our estimates of genome-wide diversity. Five additional reference populations (N = 80) from Tunisia (Tunisian native breed, N = 23), Burkina Faso (Sahel, N = 15; Djallonké, N = 12), Switzerland (Saanen, N = 15) and Romania (Carpathian, N = 15) were also included in the study for comparative purposes. We chose these five populations because DNA samples were available at the time of the investigation. Total genomic DNA was purified by using a standard phenol–chloroform extraction protocol, as described elsewhere [9].

Following the instructions of the manufacturer [10], we genotyped these 256 goat samples with the Goat SNP50 BeadChip, which features 52,295 SNPs that are distributed across the whole caprine genome [7, 10]. Sample and marker-based quality control measures were performed with the GenomeStudio software (Illumina) by setting the GenCall score cutoff to 0.15 and an average call rate of 99 %. GenomeStudio was also used to generate PLINK [11] input files to filter out non-informative SNPs: unmapped SNPs, SNPs that mapped to the X-chromosome and SNPs with minor allele frequencies (MAF) lower than 0.05 or that did not adjust to the Hardy–Weinberg expectation (*P* value ≤0.001). In addition, we performed a moderate linkage disequilibrium (LD) pruning step with the command—*indep 50 5 2* of the PLINK software, as recommended in the PLINK manual [11] and in a previous publication [12]. This is necessary because stretches of SNPs with a low MAF and genomic regions with many SNPs and strong LD are prone to erroneous identification of autozygous ROH [12]. After these filtering steps, 39,257 SNPs were available for the genetic analyses. The goat genome assembly CHI_1.0 [13] was used as a reference. Chromosome lengths, number of SNPs per chromosome and average and minimum distances between SNPs are in Additional file 3: Table S2.

### Population genetics analyses

PLINK [11] was used to calculate the observed (H_o_) and expected (H_e_) heterozygosities for each population, whereas pairwise F_ST_ values were obtained with Arlequin 3.5 [14]. Population structure was inferred with two different programs: PLINK, which applies standard classical multidimensional scaling (MDS) plot analysis based on a matrix of genome-wide pairwise identity-by-state distances, and ADMIXTURE v. 1.23 [15], which calculates maximum likelihood estimates of individual ancestries based on data provided by multiple loci.

PLINK [11] was also used to identify runs of homozygosity (ROH) by running a sliding window that scans the genomic distribution of SNP data to identify stretches of homozygous SNPs. With PLINK, we calculated the fraction of 50 SNP-windows that are almost completely homozygous i.e. one heterozygous SNP, five missing genotypes and a maximum gap of 1000 kb were allowed. We used this approach, instead of just considering windows of SNPs that are completely homozygous, because it increases the power of detecting truly autozygous segments, especially when handling high-density SNP data [12]. Moreover, this strategy is particularly suitable for livestock populations because they have much higher levels of autozygosity than model organisms and thus identification of longer ROH is expected [12, 16].

## Results

### Estimates of whole-genome diversity and analysis of population structure

**Fig. 1:**
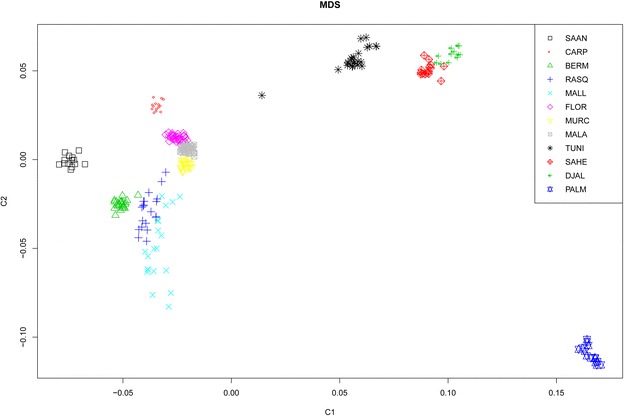
Multidimensional scaling plot for 12 caprine populations. Caprine populations analysed: Spain (Bermeya, Blanca de Rasquera, Malagueña, Murciano-Granadina, Florida, Mallorquina and Palmera), Tunisia, Burkina Faso (Sahel and Djallonké), Romania (Carpathian) and Switzerland (Saanen). This analysis is based on genome-wide identity-by-state pairwise distances calculated with the PLINK software [11] on the basis of 39,257 SNPs. Three main clusters are observed: (1) Spanish and European breeds, (2) African breeds, and (3) the Palmera breed from the Canary Islands

Descriptive statistics of genetic diversity and differentiation are in Additional file 4: Table S3. Expected and observed heterozygosities estimated in Spanish, African and European (Saanen and Carpathian) breeds were similar, with average H_o_ values of 0.38, 0.38 and 0.42, respectively. Only the Palmera breed had a lower level of genetic diversity, with H_o_ and H_e_ values less than 0.29. The MDS in Fig. 1 shows that the Spanish breeds are more closely related to Saanen and Carpathian goats than to breeds from Tunisia and Burkina Faso (Sahel and Djallonké). Within Spain, breeds also clustered according to their geographic origin, i.e. we observed: a tight Southern Spanish cluster that included the Florida, Malagueña and Murciano-Granadina breeds; a more scattered Northern Spanish cluster, including the Blanca de Rasquera, Bermeya and Mallorquina breeds was found close to the “Southern” cluster; the Palmera breed formed another highly differentiated group; and goats from Tunisia and Burkina Faso were positioned far from the Spanish breeds (except for one Tunisian sample) and displayed moderate levels of genetic differentiation.

The results of the admixture analysis agreed well with the findings outlined above (see Additional file 5: Figure S2). We observed, at K = 2, a strong genetic differentiation between the Palmera breed and the remaining populations and this was also found at higher K-values. At K = 3, clearly the genetic background that predominates in African goats (colored in green) is present in Spanish Peninsular and Balearic breeds (16.5 % on average) although their ancestry is essentially European (blue background, 79.4 %). This putative African genetic background was particularly evident in the Southern Spanish breeds (25.2 %), which is consistent from a geographic point of view, and also in Carpathian goats (23.7 %). At higher K-values (K = 5 and 6), the Florida and Mallorquina breeds had distinct genetic backgrounds while the remaining Iberian populations showed a mixed ancestry (see Additional file 5: Figure S2), which suggested that multiple gene pools were involved in their foundation.

**Fig. 2:**
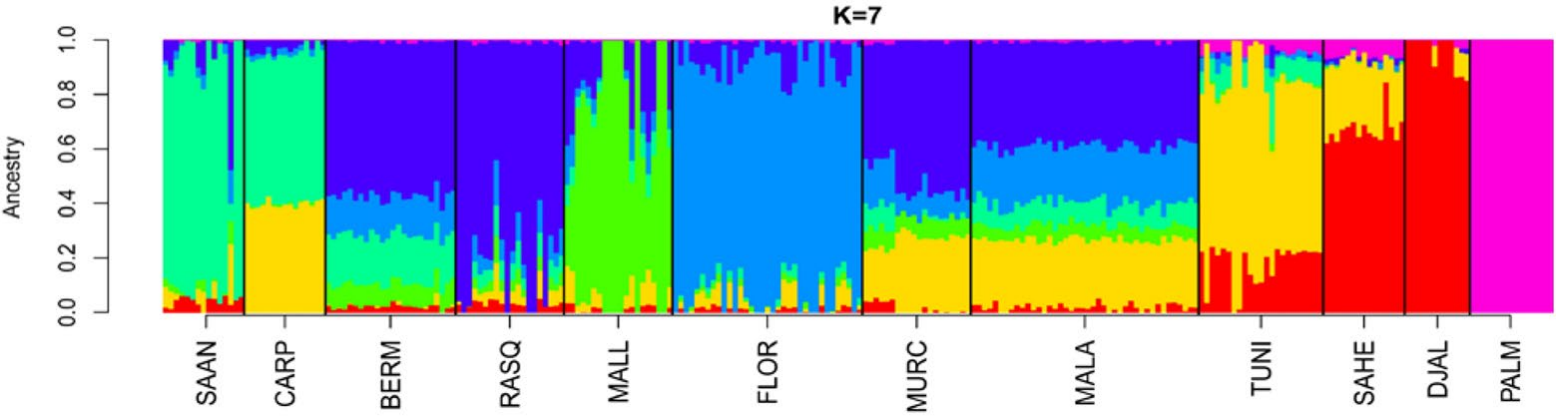
Maximum likelihood estimation of individual ancestries calculated with the admixture software. We assumed a K-value 7 because it had the lowest cross-validation error. We took into consideration data from 39,257 SNPs used to genotype 12 caprine populations from Spain (Bermeya, Blanca de Rasquera, Malagueña, Murciano-Granadina, Florida, Mallorquina and Palmera), Tunisia, Burkina Faso (Sahel and Djallonké), Romania (Carpathian) and Switzerland (Saanen). This analysis shows that the Southern Spanish breeds, Malagueña and Murciano-Granadina, together with the Carpatian breed from Romania show evidence of potential introgression with North African (Tunisian) goats

The analysis at K = 7 (Fig. 2), i.e. the K-value that showed the lowest cross-validation error, confirmed the results described above. The genetic background (shown in orange in Fig. 2) that predominates in Tunisian goats (68.2 %) was also present in the Southern Spanish Malagueña and Murciano-Granadina breeds (25.1 %) and, as previously noted, in Carpathian goats (39.8 %). In contrast, we could not find any significant evidence of a Burkina Faso ancestry for Spanish breeds. The results obtained at K-values higher than 7 supported the main trends, which were highlighted in the previous analyses, and are not further discussed.

### Analysis of runs of homozygosity

**Fig. 3:**
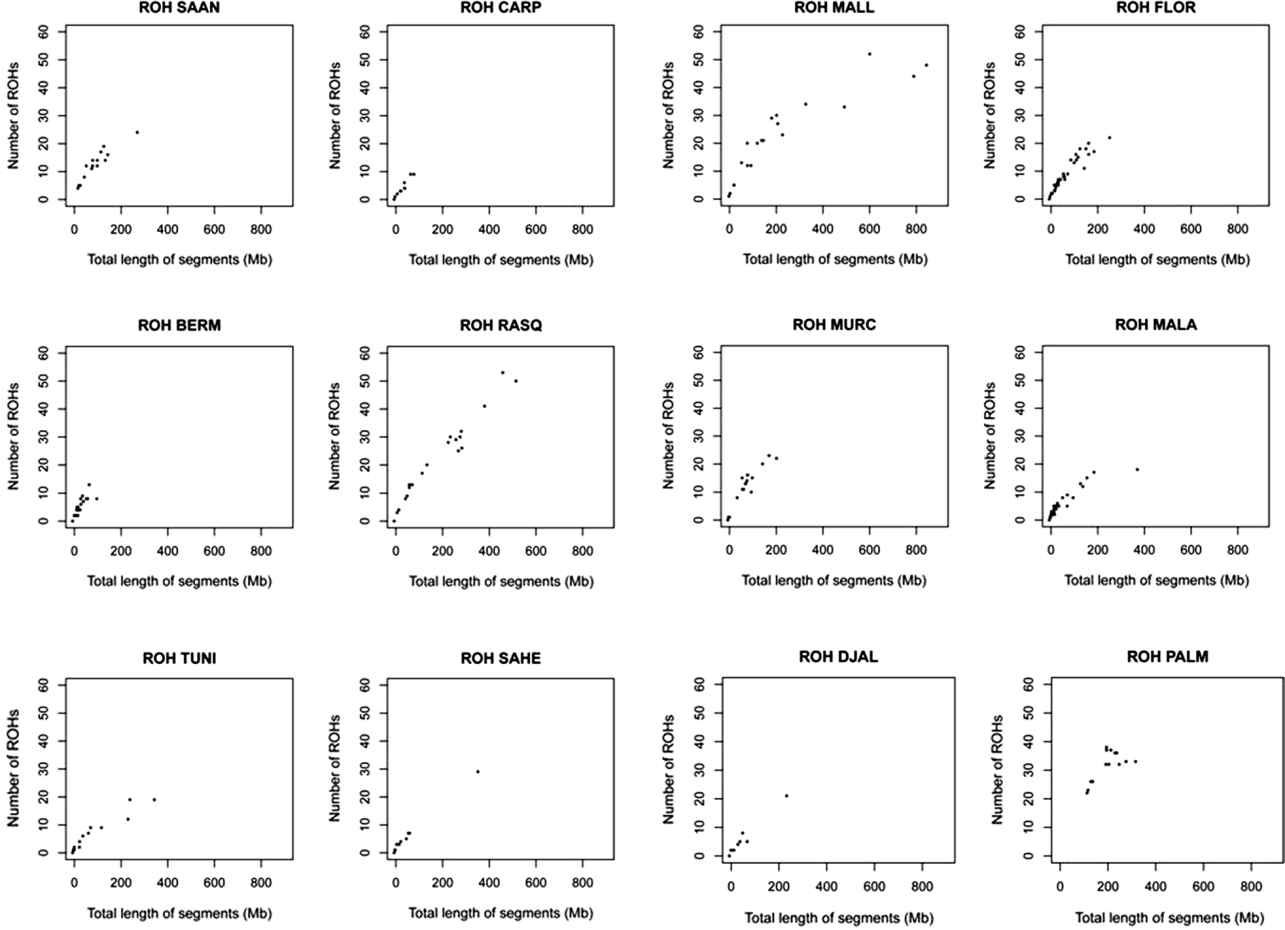
Runs of homozygosity (ROH) identified for 12 caprine populations. The number of ROH found for each individual genome (*y-axis*) is plotted against ROH total size (i.e. the number of Mb covered by ROH in each genome, *x-axis*). We analysed goats from Spain (Bermeya, Blanca de Rasquera, Malagueña, Murciano-Granadina, Florida, Palmera and Mallorquina), Tunisia, Burkina Faso (Sahel and Djallonké), Romania (Carpathian) and Switzerland (Saanen). For the Mallorquina and Blanca de Rasquera breeds, we observed many individuals with a large number of ROH (>40) and that a substantial fraction of the genome is covered by ROH (>400 Mb). In contrast, for the Palmera breed, the number of ROH is relatively large (20 to 40) but the fraction of the genome covered by ROH is quite small (<400 Mb)

The abundance, length and genomic distribution of ROH constitute a valuable source of information about the demographic history of livestock species [17]. Our study demonstrated that the average size of ROH was around 6.18 Mb (see Additional file 6: Table S4), with a size range from 4.9 Mb (Sahel) to 8.4 Mb (Mallorquina). Besides, we were able to identify three main distribution patterns (Fig. 3). In the most common distribution pattern, the majority of the individuals clustered close to the origin of coordinates because each one carried 0 to 20 ROH with a total length less than 200 Mb. The second category, that was featured by the Mallorquina and Blanca de Rasquera breeds, included individuals that carried a large number of ROH (30 to 50 ROH per individual, with a total length of 600 to 800 Mb) and individuals with a much smaller number of ROH (thus matching the first category defined above). The third category was represented exclusively by the Palmera breed for which all individuals had between 20 and 40 ROH with a total length between 100 and 400 Mb.

**Fig. 4:**
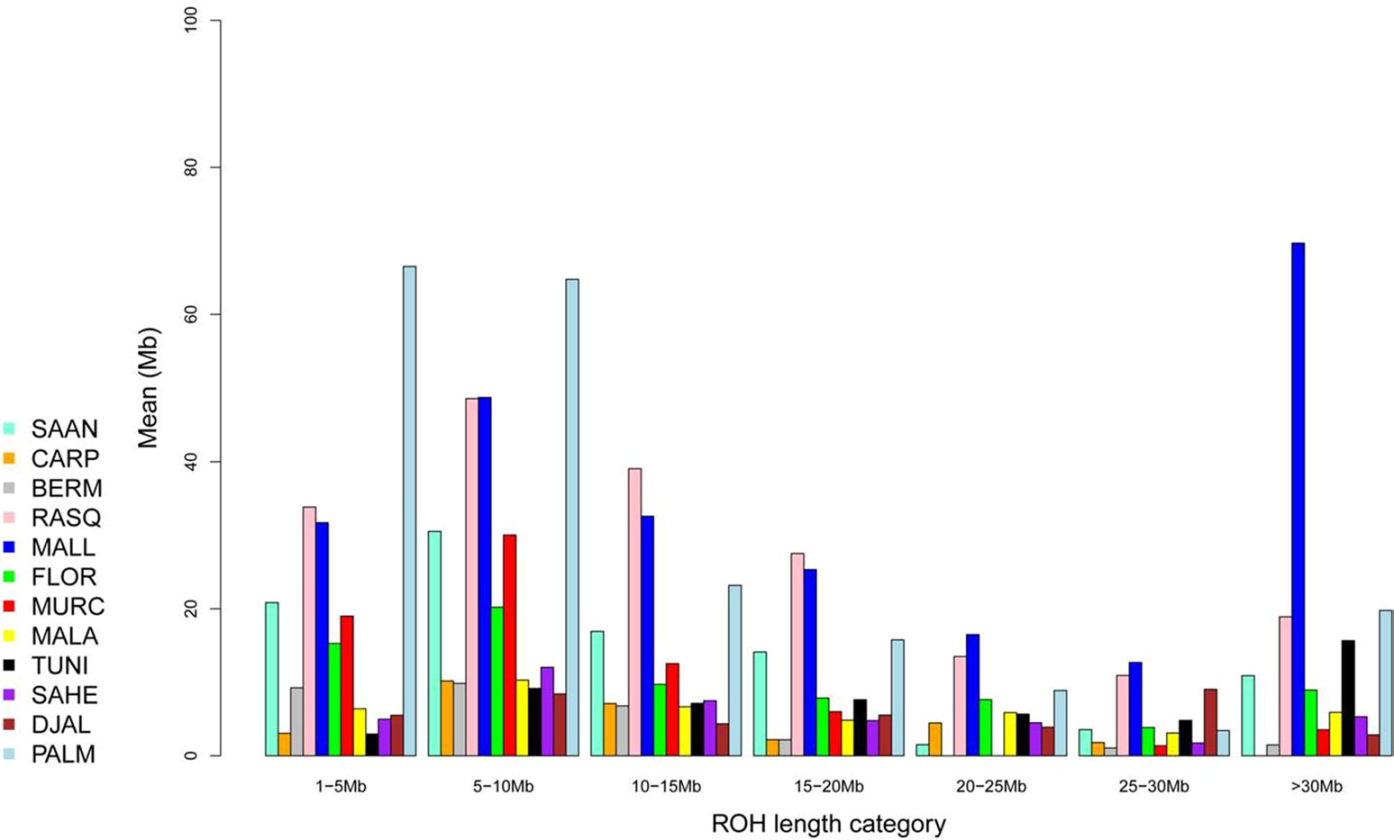
Classification of ROH in seven categories (*x-axis*) according to size (from 1 to 5 Mb to more than 30 Mb) and mean sum of ROH (*y-axis*, measured in megabases) within each ROH category and averaged per breed. We analysed 12 caprine populations from Spain (Bermeya, Blanca de Rasquera, Malagueña, Murciano-Granadina, Florida, Palmera and Mallorquina), Tunisia, Burkina Faso (Sahel and Djallonké), Romania (Carpathian) and Switzerland (Saanen). This figure shows that for the Mallorquina breed, the mean sum of ROH with sizes greater than 30 Mb is several times larger than that for the remaining breeds. In contrast, in Palmera goats, ROH with sizes between 5 to 10 and 10 to 15 Mb display the largest mean sums (>60 Mb for each category)

Analysis of the distribution of ROH according to their size (Fig. 4) showed that, for the Palmera breed, ROH that were shorter than 10 Mb predominated. A similar, although less obvious, pattern was detected for the Blanca de Rasquera population. In contrast, a bimodal distribution with extremely long (>30 Mb) and relatively short (<10 Mb) ROH was found in the Mallorquina breed. For the remaining breeds, ROH size distribution was relatively uniform, although, in general, long ROH were less abundant than medium-long or small ROH.

## Discussion

### About the high levels of autosomal diversity detected in Spanish goats

In 2005, Pereira et al. [18] characterized the mitochondrial variation of Portuguese goats and observed a high level of genetic diversity within this population in spite of the fact that it is located at the periphery of the goat geographic distribution in the Old World. These results were recently confirmed by genotyping a wide array of Iberian goat breeds with a panel of microsatellites [6]. The results of our high-throughput autosomal analysis are consistent with those reported in [6] (see Additional file 4: Table S3) i.e. average H_o_ and H_e_ values were around 0.38 (H_o_ = 0.29 to 0.42 and H_e_ = 0.28 to 0.41). These results are also similar to those from a genome-wide survey of Italian goats [19] and a worldwide analysis of sheep variation [20]. Expected heterozygosity values of sheep breeds from South and West Europe ranged from 0.22 to 0.38, with an average value of 0.35. The high level of genetic diversity found in South European sheep breeds may reflect the first wave of dissemination of the Neolithic package along the Mediterranean basin [20]. Another explanation could be related with the extended practice of transhumance in Southern Europe. For instance, during the eighteenth century, the number of sheep, cattle and goats that followed the Spanish transhumance pathways reached ~3.7 million. Moving from the winter meadows to the summer pastures (and viceversa), they often covered distances of hundreds of kilometers, which favoured genetic exchanges among populations from distant regions. Still today, around 450,000 sheep, 60,000 cattle and 70,000 goats are herded across the Spanish network of transhumance tracks, which allows the adaptation of livestock production in marginal areas to seasonal variations in weather conditions and land productivity.

The levels of genetic diversity that we found in breeds with large and expanding population sizes, such as Murciano-Granadina (current census N ~ 100,000, H_e_ = 0.39) and Malagueña (N ~ 46,000, H_e_ = 0.41) were quite similar to those found for much smaller endangered populations, such as Bermeya (N ~ 2000, H_e_ = 0.40) and Blanca de Rasquera (N ~ 5000, H_e_ = 0.38). This unexpected observation could be due to a technical bias i.e. that only the SNPs with intermediate frequencies, in a representative sample of goat breeds, were selected to form part of the Goat SNP50 BeadChip [7, 10]. Similarly, the microsatellites that have been used in population genetic studies were not chosen at random but were selected on the basis of a high level of polymorphism in distinct populations. This results in an upward bias of the diversity parameters that are estimated on the basis of microsatellites and SNPs genotypes. It is likely that such parameters inferred from whole-genome sequencing data, which are unbiased, would correlate much better with the effective sizes of the populations under study.

Introgression with African goats could be a potential source of diversity for Iberian livestock breeds [18]. Indeed, mitochondrial analysis of Canarian and Balearic goats showed that haplogroup G, which so far was exclusively found in goats from Africa and the Middle East, segregates in the Mallorquina breed [4]. Nonetheless, admixture analysis (Fig. 2) and (see Additional file 5: Figure S2) showed that, among the Spanish populations, only the Andalusian breeds, Murciano-Granadina and Malagueña, showed signs of African admixture. Regardless of the K-value under consideration, this result was particularly consistent for the Malagueña breed (see Additional file 5: Figure S2). At the most significant K-value (Fig. 2), the genetic background that predominates in Tunisian goats (indicated in orange) was also found in the Malagueña (24.9 %) and Murciano-Granadina (25.3 %) goats and was almost absent in the caprine breeds from Northern Spain (Bermeya and Blanca de Rasquera), the Balearic Islands (Mallorquina) or Switzerland (Saanen). The identification of a Tunisian genetic background in Carpathian goats (Fig. 2) was quite unexpected and may reflect introgression of this Romanian population with an exotic breed that carries African alleles. Indeed, Murciano-Granadina goats have been exported to Romania [21], which may have led to crossbreeding with Carpathian individuals. Finally, we did not observe any relationship between Palmera goats and those from the Iberian Peninsula and Africa. The high genetic divergence of Palmera goats has been interpreted as a consequence of a strong founder effect and prolonged geographic isolation [6].

Our study provides preliminary evidence of the presence of a North African genetic background in Andalusian goats (Fig. 2). This result is consistent with previous data that showed that bovine breeds from Southern Europe have been significantly introgresssed (~19 %) with cattle of African origin [8]. Besides, a large-scale mitochondrial and Y-chromosome analysis of European, Asian and African breeds identified traces of gene flow between goats from the Maghreb and the Iberian Peninsula [22]. The existence of genetic connections between Spanish and North African goats is consistent with the close geographic distance between these two geographic areas (14.3 km at the narrowest point of the Gibraltar Strait). Besides, Andalusia was occupied by Berber and Arab troops from the 8th to the 15th centuries during which there was ample opportunity to introduce African livestock in the Iberian Peninsula. Indeed, the Moorish invasion of Spain involved the introduction of new crops, such as sugar cane, cotton and aubergine that were subsequently exported to other European locations [23]. It is even possible that genetic exchanges between Spanish and African domestic animals occurred in much more ancient times. Indeed, a mitochondrial analysis of bovine archaeological remains from the Portalón Cave at Atapuerca suggested that African bovines may have entered the Iberian Peninsula during the Bronze Age [24]. In this regard, a coalescent analysis of microsatellite data from African and Spanish goats provided support for the existence of a significant historical gene flow between caprine populations on both sides of the Gibraltar Strait [25].

### The distribution of runs of homozygosity in the genomes of Spanish goats closely matches breed demography

In general, we identified ROH with an average length of 6.28 Mb, but their sizes varied considerably, as reflected by the standard deviations in See Additional file 6: Table S4. Besides, ROH that ranged from 5 to 10 Mb tended to be the most abundant ones (Fig. 4). In contrast, other studies on cattle and pigs reported much shorter ROH. For example, analysis of the distribution of ROH lengths among nine cattle breeds showed that small ROH between 1 and 5 Mb were much more frequent than those that were longer than 5 Mb [26]. Similarly, the majority of the ROH that were detected in five Italian cattle breeds were 1 to 2 Mb long [27]. This discrepancy may be due to a technological issue. Purfield et al. [26] compared the efficiency of the 770 versus the 50 K Bovine SNP BeadChip for the identification of ROH and found that 157,600 and 19,078 ROH were detected with the SNP770 and the SNP50 chips, respectively. Thus, the values for total ROH length and abundance that are reported in our work are probably underestimated because a considerable amount of ROH remains undetected when using a medium-density SNP panel. Another problem associated with the use of medium-density chips is that they are more effective in detecting long than short ROH, and thus this introduces an important bias in the distribution of ROH length. Indeed, only 27.7 % of the 1 to 5 Mb long ROH that were reported for bovine breeds with the SNP770 chip were also identified with the SNP50 chip, while this percentage increased to 80 % when ROH longer than 5 Mb were considered [26].

In general, average ROH lengths were shorter in African (5.23 Mb) than in Spanish (6.68 Mb) breeds (see Additional file 6: Table S4). Similar findings were obtained by Purfield et al. [26] in a study that compared African and European cattle. As pointed out by these authors [26], this result can be explained by the important role that the concept of breeds has in Europe but not in Africa. During the last century, local types of goats were established as breeds in Europe. Breeds are, in principle, closed populations whose genealogies are registered in herdbooks and that display distinctive phenotypic features that are defined in the racial standard. The very concept of breed is associated with restricted gene flow, phenotypic uniformity and increased inbreeding. This is exacerbated in certain European breeds, such as Saanen, by the widespread use of artificial insemination and intensive selection. In contrast, in Africa, the notion of breed and controlled mating are much looser, or even inexistent, and goats are usually raised under an extensive regime. These practices favour the occurrence of genetic exchanges and reduce the impact of factors that increase genomic homozygosity.

In Spanish goats, we observed a close relationship between the overall number and length of ROH (Figs. 3, 4) and demographic history. For most of the breeds, a limited number of ROH (between 0 and 20 per individual) were detected and covered less than 200 Mb. This finding reflects that ancient and recent inbreeding have had a weak impact on the genomes of most of the Spanish (and African) breeds analysed here. In contrast, none of the Palmera goats that we examined had less than 20 ROH and these covered a substantial part of their genomes (200 to 400 Mb) and were relatively short (<10 Mb). Previous studies demonstrated that Palmera goats have a decreased mitochondrial and autosomal variability when compared to other Spanish breeds [6, 28]. These features are consistent with a strong and ancient founder effect combined with a small historical effective size, probably as a consequence of geographic isolation. The Palmera breed has a current census of ~7600 individuals, but previous genetic analyses indicated that they probably descend from a few founder goats brought by the Imazighen people who settled in the Canary Islands 2500 YBP [4, 28]. Moreover, Palmera goats remained geographically isolated during two millenia, until the Canarian archipelago was conquered by the Castile Crown in the fifteenth century. This might be the main factor that explains the strong genetic identity and reduced genetic variation of this insular population (Figs. 1, 2) and (see Additional file 4: Table S3).

A different pattern of ROH distribution was detected for the Mallorquina and Blanca de Rasquera breeds, which comprise individuals that carry a large number of long ROH (Fig. 3). This pattern, which is typically produced by recent inbreeding, was particularly evident in the Mallorquina breed, for which the most abundant ROH were longer than 30 Mb (Fig. 4). This finding was expected because the Mallorquina breed has a census of 58 breeding males and 169 breeding females that are distributed on 15 farms, which means that it is at the verge of extinction. The situation of the Blanca de Rasquera breed is less critical because it has a much larger census (around 5000 individuals). However, this breed is experiencing a strong demographic decline (80 % reduction in population size during the last 50 years) because of the progressive abandonment of low income rural activities and competition with more productive exotic breeds [29].

The accumulation of long ROH in individuals of the Mallorquina and the Blanca de Rasquera breeds could have consequences on the biological fitness of these populations. Long ROH are enriched in genomic regions that carry deleterious mutations and there is a strong linear relationship between the genomic fraction of ROH and the number of individuals that carry deleterious homozygous mutations [30]. For example, a large number of genomic regions that contain long ROH (>60 SNPs or >3.5 Mb) were shown to have unfavourable associations with milk yield in Holstein cattle, probably as a consequence of inbreeding depression [31].

## Conclusions

Our data illustrate how admixture, drift and demography have shaped the genome-wide diversity of Spanish goats. The results of the genome-wide SNP analyses are consistent with previous mitochondrial and microsatellite-based studies i.e. we have detected a high level of genetic diversity and a moderate population structure in Spanish goat breeds, and we have found evidence of admixture with African goats. In addition, we provide a first picture of the genomic features (number and length) of the ROH present in Spanish goat breeds. We also demonstrate that these ROH features reflect demographic history much better than simple diversity statistics (e.g. observed and expected heterozygosities).

## Additional files

10.1186/s12711-016-0229-6 Geographic location and sample size (N) and composition of the seven Spanish breeds analysed in our study.
10.1186/s12711-016-0229-6 Geographic distribution of the Spanish goat breeds under analysis.
10.1186/s12711-016-0229-6 Number of SNPs per chromosome and average, minimum and maximum distances between SNPs.
10.1186/s12711-016-0229-6 Diversity parameters: averaged observed and expected heterozygosities.
10.1186/s12711-016-0229-6 Admixture analysis of goat populations from Spain (Bermeya, Blanca de Rasquera, Malagueña, Murciano-Granadina, Florida and Mallorquina), Tunisia, Burkina Faso (Sahel and Djallonké), Romania (Carpathian) and Switzerland (Saanen).
10.1186/s12711-016-0229-6 Average ROH sizes (kb) for 12 goat populations.
