# Supplementary material for: A genome-wide perspective about the diversity and demographic history of seven Spanish goat breeds
Source: Genet Sel Evol. 2016 Jul 25;48:52. doi: 10.1186/s12711-016-0229-6 (PMC4960707; doi:10.1186/s12711-016-0229-6)
Supplement: Supplementary file 1 — 10.1186/s12711-016-0229-6 Geographic location and sample size (N) and composition of the seven Spanish breeds analysed in our study. [file 12711_2016_229_MOESM1_ESM.doc]

Table S1. Geographic location and sample size (N) and composition of the seven Spanish breeds analysed in the current study

| **Category** | **Breed** | **N** | **Location** | **Number of farms** |
| --- | --- | --- | --- | --- |
| Spanish breeds of Promotion | Murciano-Granadina | 20 (2 males) | Andalusia | 3 |
|  | Malagueña | 42 (16 males) | Andalusia | 13 |
|  | Palmera | 15 (0 males) | La Palma Island | 5 |
| Spanish breeds of Special Protection | Bermeya | 24 (5 males) | Asturias | 13 |
|  | Blanca de Rasquera | 20 (14 males) | Catalonia | 5 |
|  | Mallorquina | 20 (2 males) | Majorca Island | 5 |
|  | Florida | 35 (0 males) | Andalusia | 2 |
