# Supplementary material for: A genome-wide perspective about the diversity and demographic history of seven Spanish goat breeds
Source: Genet Sel Evol. 2016 Jul 25;48:52. doi: 10.1186/s12711-016-0229-6 (PMC4960707; doi:10.1186/s12711-016-0229-6)
Supplement: Supplementary file 2 — 10.1186/s12711-016-0229-6 Geographic distribution of the Spanish goat breeds under analysis. [file 12711_2016_229_MOESM2_ESM.ppt]

## Slide 1
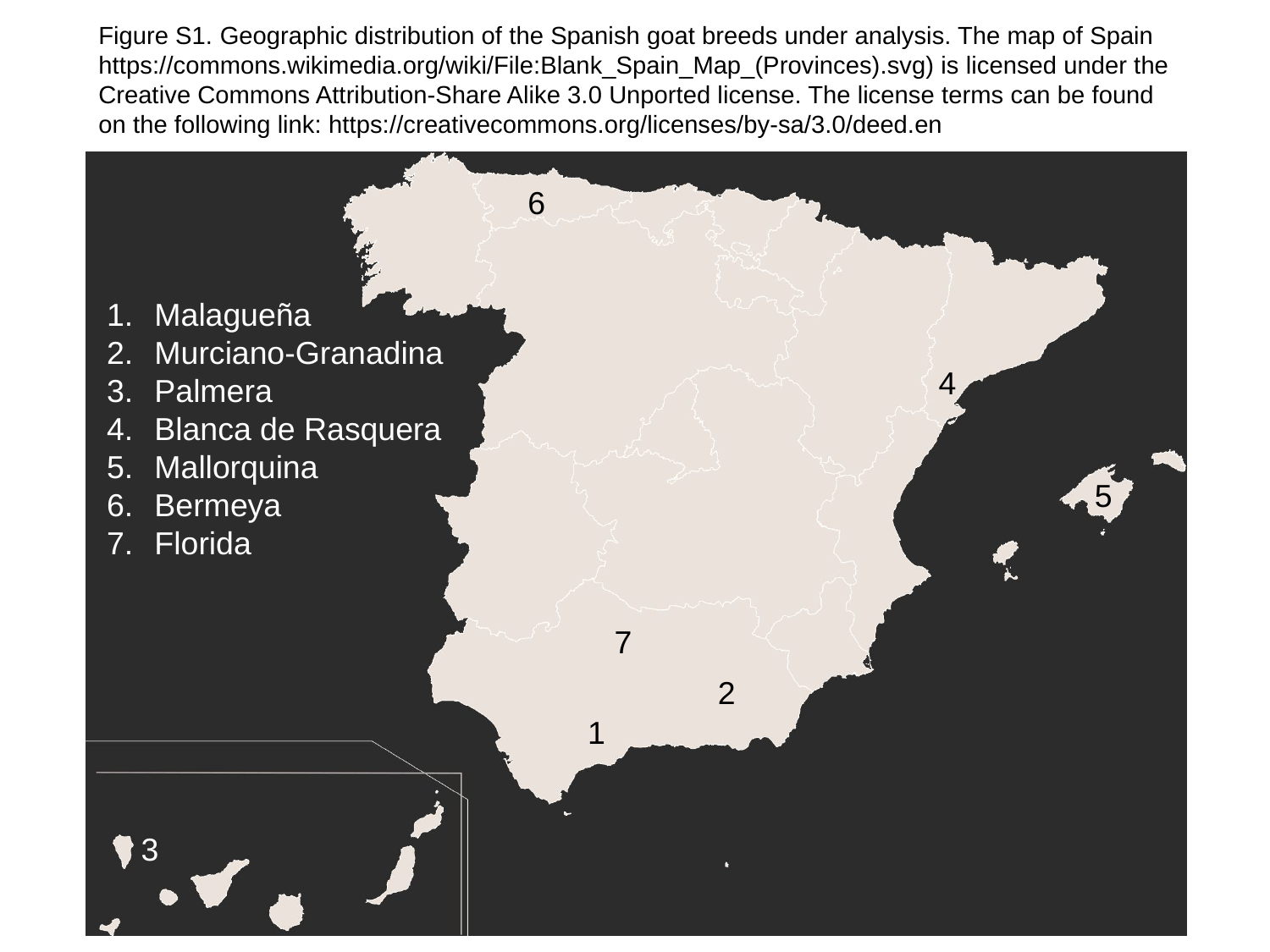

Figure S1. Geographic distribution of the Spanish goat breeds under analysis. The map of Spain https://commons.wikimedia.org/wiki/File:Blank_Spain_Map_(Provinces).svg) is licensed under the Creative Commons Attribution-Share Alike 3.0 Unported license. The license terms can be found on the following link: https://creativecommons.org/licenses/by-sa/3.0/deed.en
6
Malagueña
Murciano-Granadina
Palmera
Blanca de Rasquera
Mallorquina
Bermeya
Florida
4
5
7
2
1
3
