# Supplementary material for: A genome-wide perspective about the diversity and demographic history of seven Spanish goat breeds
Source: Genet Sel Evol. 2016 Jul 25;48:52. doi: 10.1186/s12711-016-0229-6 (PMC4960707; doi:10.1186/s12711-016-0229-6)
Supplement: Supplementary file 4 — 10.1186/s12711-016-0229-6 Diversity parameters: averaged observed and expected heterozygosities. [file 12711_2016_229_MOESM4_ESM.doc]

| **Population** | **Averaged H_o_** | **Averaged H_e_** | **Pairwise F_ST_** | | | | | | | | | | |
| --- | --- | --- | --- | --- | --- | --- | --- | --- | --- | --- | --- | --- | --- |
| Saanen | 0.419 | 0.403 | - |  |  |  |  |  |  |  |  |  |  |
| Carpathian | 0.426 | 0.413 | 0.050 | - |  |  |  |  |  |  |  |  |  |
| Bermeya | 0.406 | 0.394 | 0.063 | 0.065 | - |  |  |  |  |  |  |  |  |
| Blanca de Rasquera | 0.380 | 0.382 | 0.078 | 0.075 | 0.048 | - |  |  |  |  |  |  |  |
| Mallorquina | 0.368 | 0.371 | 0.092 | 0.089 | 0.064 | 0.075 | - |  |  |  |  |  |  |
| Florida | 0.409 | 0.391 | 0.081 | 0.072 | 0.054 | 0.064 | 0.077 | - |  |  |  |  |  |
| Murciano-Granadina | 0.404 | 0.392 | 0.071 | 0.065 | 0.045 | 0.055 | 0.067 | 0.054 | - |  |  |  |  |
| Malagueña | 0.417 | 0.412 | 0.059 | 0.048 | 0.033 | 0.043 | 0.056 | 0.036 | 0.031 | - |  |  |  |
| Tunisian | 0.405 | 0.400 | 0.096 | 0.059 | 0.082 | 0.084 | 0.093 | 0.070 | 0.064 | 0.048 | - |  |  |
| Sahel | 0.374 | 0.364 | 0.128 | 0.102 | 0.099 | 0.102 | 0.112 | 0.087 | 0.082 | 0.066 | 0.043 | - |  |
| Djallonké | 0.352 | 0.340 | 0.150 | 0.127 | 0.121 | 0.126 | 0.136 | 0.111 | 0.107 | 0.090 | 0.073 | 0.041 | - |
| Palmera | 0.289 | 0.279 | 0.220 | 0.196 | 0.191 | 0.197 | 0.207 | 0.180 | 0.180 | 0.156 | 0.146 | 0.153 | 0.186 |

Table S3. Diversity parameters in 12 goat breeds. Averaged observed and expected heterozygosities are indicated as H_o_ and H_e_, respectively.
