# Supplementary material for: A genome-wide perspective about the diversity and demographic history of seven Spanish goat breeds
Source: Genet Sel Evol. 2016 Jul 25;48:52. doi: 10.1186/s12711-016-0229-6 (PMC4960707; doi:10.1186/s12711-016-0229-6)
Supplement: Supplementary file 5 — 10.1186/s12711-016-0229-6 Admixture analysis of goat populations from Spain (Bermeya, Blanca de Rasquera, Malagueña, Murciano-Granadina, Florida and Mallorquina), Tunisia, Burkina Faso (Sahel and Djallonké), Romania (Carpathian) and Switzerland (Saanen). [file 12711_2016_229_MOESM5_ESM.pdf]

Figure S2. Admixture analysis (K = 2-12) of goats from Spain (Bermeya, Blanca de Rasquera, Malagueña, Murciano-Granadina, Florida and Mallorquina), Tunisia, Burkina Faso (Sahel and Djallonké) and Central East Europe (Carpathian and Saanen).

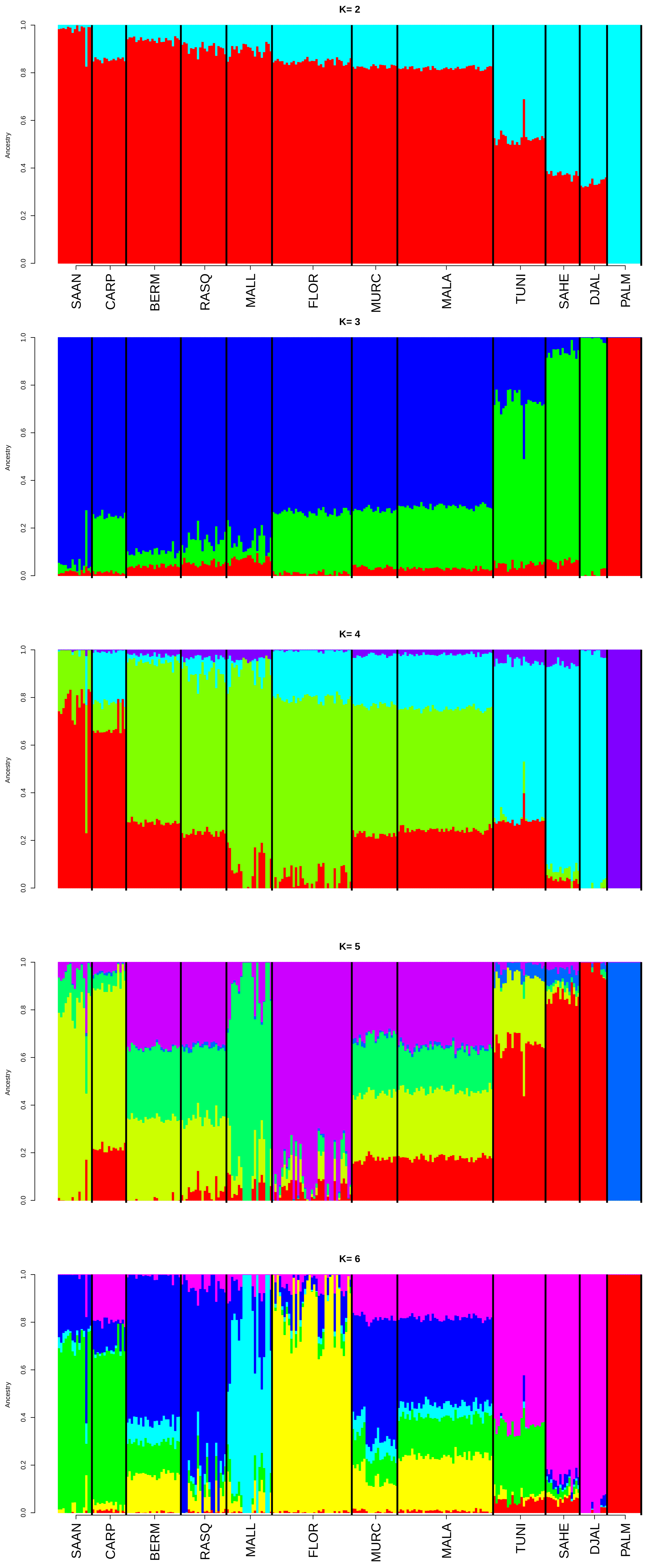
K= 2
K= 3
K= 4
K= 5
K= 6
Ancestry
0.0
0.2
0.4
0.6
0.8
1.0
SAAN
CARP
BERM
RASQ
MALL
FLOR
MURC
MALA
TUNI
SAHE
DJAL
PALM

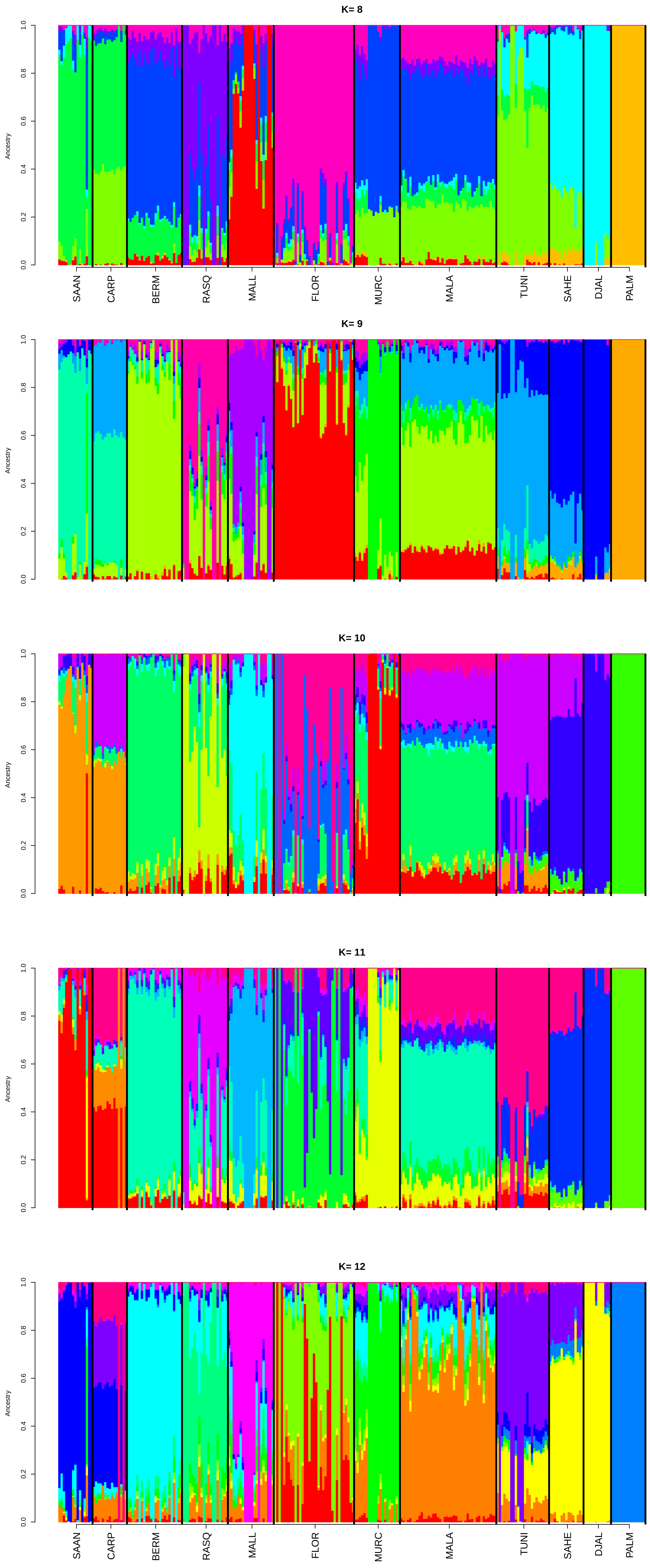
K= 8
K= 9
K= 10
K= 11
K= 12
Ancestry
0.0
0.2
0.4
0.6
0.8
1.0
SAAN
CARP
BERM
RASQ
MALL
FLOR
MURC
MALA
TUNI
SAHE
DJAL
PALM
